# Supplementary material for: Sexually Dimorphic Gene Expression in X and Y Sperms Instructs Sexual Dimorphism of Embryonic Genome Activation in Yellow Catfish (Pelteobagrus fulvidraco)
Source: Biology (Basel). 2022 Dec 14;11(12):1818. doi: 10.3390/biology11121818 (PMC9775105; doi:10.3390/biology11121818)

A

Sperm for WGBS	Clean Reads	Mapped Reads	Mapping Rate (%)	Uniquely Mapped Reads	Uniquely Mapping Rate (%)	Bisulfite Conversion Rate (%)	Duplication Rate (%)	Average Depth	Coverage
X1	264,558,878	211,936,914	80.11	181,536,295	68.62	99.57	13.84	31.58	86.98
X2	288,216,188	230,283,608	79.9	196,846,268	68.3	99.5	14.04	34.16	86.84
X3	281,723,878	225,557,346	80.06	192,512,872	68.33	99.59	13.96	33.44	86.96
Y1	260,669,208	209,285,906	80.29	178,096,247	68.32	99.59	15.38	30.42	86.03
Y2	198,227,002	156,922,062	79.16	134,348,068	67.77	99.61	12.67	23.7	85.29
Y3	239,269,788	189,600,565	79.24	161,463,874	67.48	99.67	10.59	29.3	85.19

Sperm for WGBS	Methylated C's in CpG context	% mCpG	Methylated C's in CHG context	% mCHG	Methylated C's in CHH context	% mCHH
X1	16,349,756	98.03%	84,021	0.504%	244,489	1.466%
X2	16,450,375	97.83%	92,929	0.553%	271,797	1.616%
X3	16,484,275	98.11%	81,941	0.488%	235,111	1.399%
Y1	16,066,293	98.07%	79,526	0.485%	236,206	1.442%
Y2	15,286,997	98.19%	71,520	0.459%	209,574	1.346%
Y3	15,686,067	98.30%	69,700	0.437%	201,055	1.260%

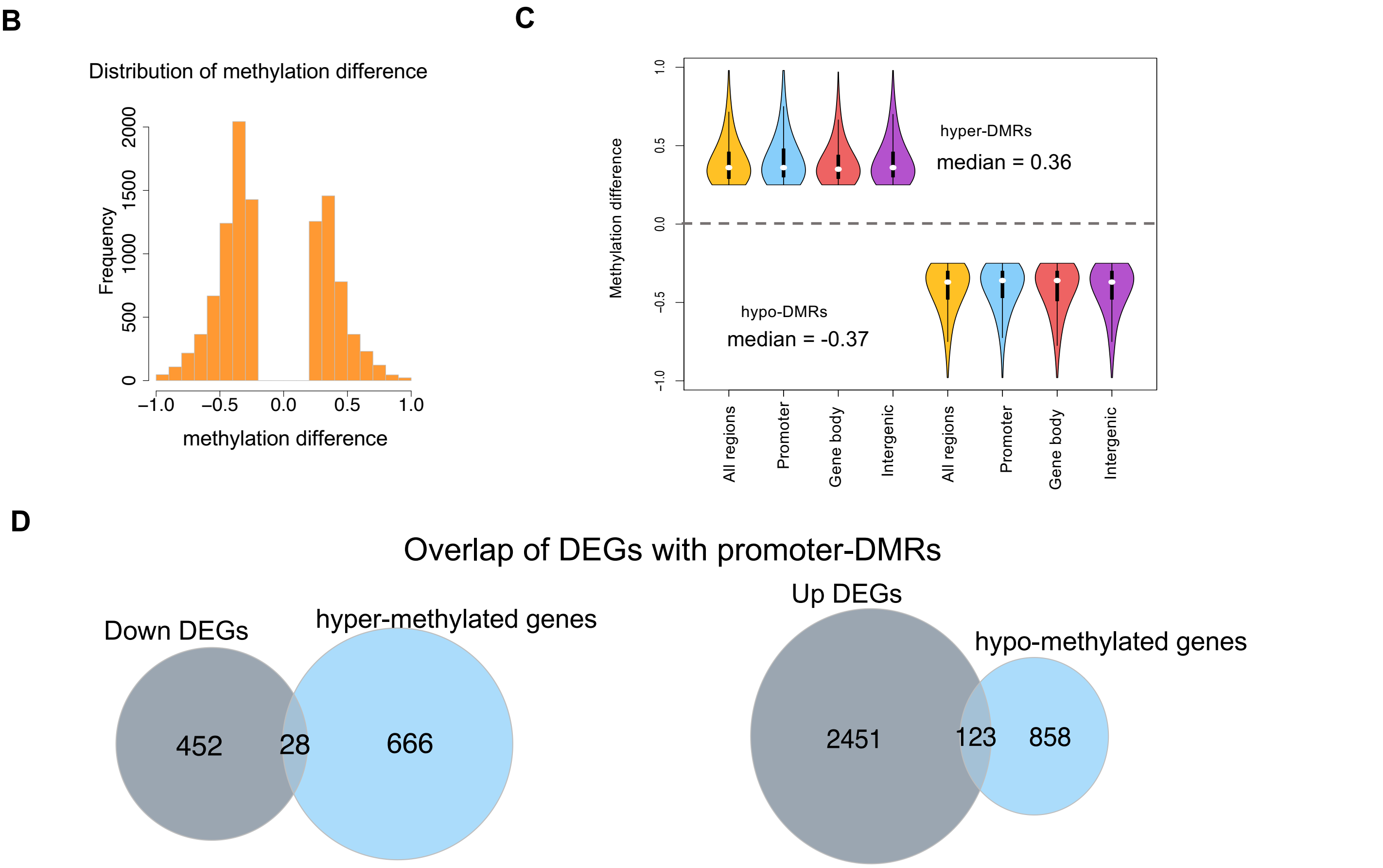

Supplement: Supplementary file 1 [file biology-11-01818-s001.zip › Figure S2.pdf]
