# Supplementary material for: Sexually Dimorphic Gene Expression in X and Y Sperms Instructs Sexual Dimorphism of Embryonic Genome Activation in Yellow Catfish (Pelteobagrus fulvidraco)
Source: Biology (Basel). 2022 Dec 14;11(12):1818. doi: 10.3390/biology11121818 (PMC9775105; doi:10.3390/biology11121818)
Supplement: Supplementary file 1 [file biology-11-01818-s001.zip › Figure S3.pdf]

A

WGRS Samples	Clean Reads	Mapped Reads	Mapping Rate (%)	Properly paired reads	Properly paired Rate (%)	Average Depth	Coverage (>0×)	Coverage (>=10×)	Coverage (>=30×)
XX female-1	127,676,371	126,714,150	99.25%	120,576,388	95.63	25.96	99.59%	98.08%	25.13%
XX female-2	137,569,436	136,562,957	99.27%	129,865,846	95.62	28.33	99.60%	98.38%	37.83%
XX female-3	170,798,775	168,473,284	98.64%	159,275,496	94.42	34.63	99.62%	98.82%	69.18%
XX neo-male-1	146,217,730	145,45,460	99.27%	137,145,182	95.07	28.83	99.61%	98.62%	43.52%
XX neo-male-2	143,865,614	142,825,504	99.28%	134,234,864	94.73	28.41	99.61%	98.62%	39.05%
XX neo-male-3	150,909,443	149,858,948	99.30%	140,019,904	94.34	29.21	99.62%	98.73%	43.20%
YY super-male-1	137,712,569	136,797,658	99.34%	128,742,742	94.93	27.48	99.58%	98.56%	35.74%
YY super-male-2	140,742,656	139,818,920	99.34%	131,831,388	95.08	28.35	99.59%	98.61%	40.49%
YY super-male-3	150,816,920	149,808,490	99.33%	141,078,302	94.96	30.22	99.60%	98.76%	50.42%

B

RNA-seq Samples	Clean Reads	Mapped Reads	Mapping Rate (%)	Uniquely Mapped Reads	Uniquely Mapping Rate (%)	Duplication Rate (%)	Average Depth	Coverage(%)
xx-2cell-1	34,764,768	33,195,452	95.49	32,366,584	93.1	21.16	16.79	54.35
xx-2cell-2	36,538,744	34,944,468	95.64	33,986,096	93.01	18.16	17.31	55.44
xx-2cell-3	37,837,959	36,162,597	95.57	34,998,948	92.5	18.42	17.84	55.92
xx-64cell-1	32,854,528	31,540,221	96.00	30,830,028	93.84	14.06	15.24	62.03
xx-64cell-2	33,748,457	32,155,264	95.28	31,182,267	92.4	13.81	15.01	62.18
xx-64cell-3	34,243,822	32,578,498	95.14	31,758,124	92.74	12.16	15.47	62.48
xx-high-1	42,984,975	41,053,286	95.51	39,950,368	92.94	11.17	18.70	66.27
xx-high-2	36,754,555	34,674,884	94.34	33,794,942	91.95	10.45	16.19	64.84
xx-high-3	39,651,873	38,143,795	96.20	36,990,785	93.29	12.73	17.53	65.37
xx-low-1	41,904,231	40,123,808	95.75	38,055,535	90.82	14.17	17.51	72.36
xx-low-2	43,523,690	41,701,174	95.81	39,388,805	90.5	13.04	18.04	73.32
xx-low-3	40,855,758	38,981,334	95.41	36,897,924	90.31	13.49	16.94	72.09
xx-bud-1	42,907,426	40,777,904	95.04	39,513,345	92.09	14.42	17.72	79.79
xx-bud-2	40,398,069	38,450,084	95.18	37,215,633	92.12	13.98	16.79	79.31
xx-bud-3	38,987,913	37,202,436	95.42	36,056,337	92.48	13.93	16.24	78.89
xy-2cell-1	41,747,062	39,887,460	95.55	38,699,519	92.7	19.57	19.35	57.06
xy-2cell-2	36,744,514	35,245,752	95.92	34,157,786	92.96	19.58	17.50	56.13
xy-2cell-3	39,954,935	37,525,716	93.92	36,341,396	90.96	19.74	18.39	57.30
xy-64cell-1	39,949,866	38,462,377	96.28	37,474,777	93.8	15.26	18.31	62.45
xy-64cell-2	38,082,421	36,866,933	96.81	35,925,071	94.34	16.10	17.64	63.80
xy-64cell-3	41,291,887	39,934,967	96.71	38,663,887	93.64	16.22	18.97	63.67
xy-high-1	42,152,969	40,832,658	96.87	39,264,210	93.15	1615	19.14	65.35
xy-high-2	40,863,542	39,523,009	96.72	37,807,981	92.52	15.85	18.46	65.65
xy-high-3	40,863,406	39,323,999	96.23	37,537,473	91.86	13.91	18.16	65.73
xy-low-1	36,862,461	35,412,005	96.07	32,956,073	89.4	15.27	15.57	71.85
xy-low-2	40,063,162	38,379,629	95.80	35,326,445	88.18	16.55	16.74	72.08
xy-low-3	41,398,339	39,598,487	95.65	36,799,384	88.89	16.10	17.06	73.25
xy-bud-1	37,290,451	35,525,256	95.27	33,777,415	90.58	20.83	15.74	77.11
xy-bud-2	38,386,332	36,555,760	95.23	34,787,526	90.62	16.35	15.97	78.59
xy-bud-3	36,494,493	34,899,730	95.63	33,040,591	90.54	15.46	15.44	78.37
