# Supplementary material for: Sexually Dimorphic Gene Expression in X and Y Sperms Instructs Sexual Dimorphism of Embryonic Genome Activation in Yellow Catfish (Pelteobagrus fulvidraco)
Source: Biology (Basel). 2022 Dec 14;11(12):1818. doi: 10.3390/biology11121818 (PMC9775105; doi:10.3390/biology11121818)
Supplement: Supplementary file 1 [file biology-11-01818-s001.zip › Figure S4.pdf]

A

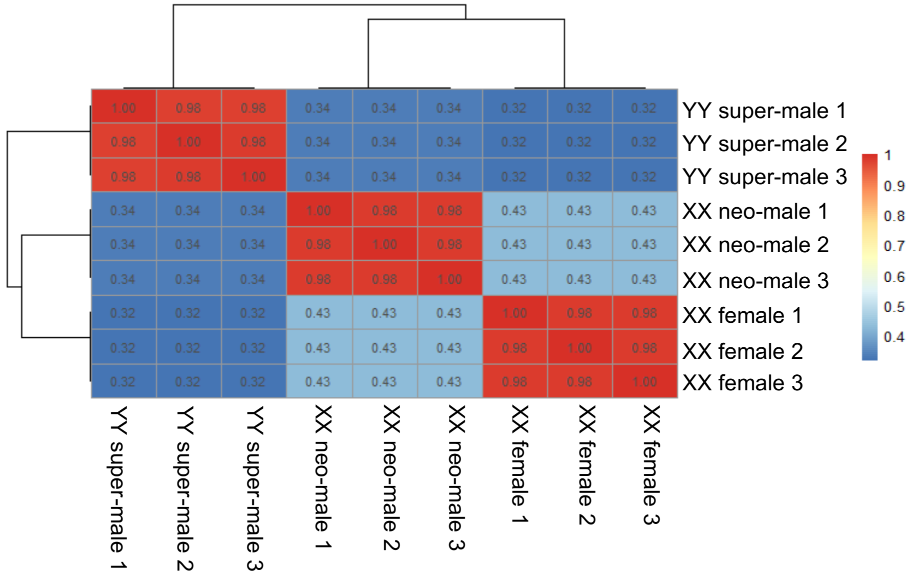

B

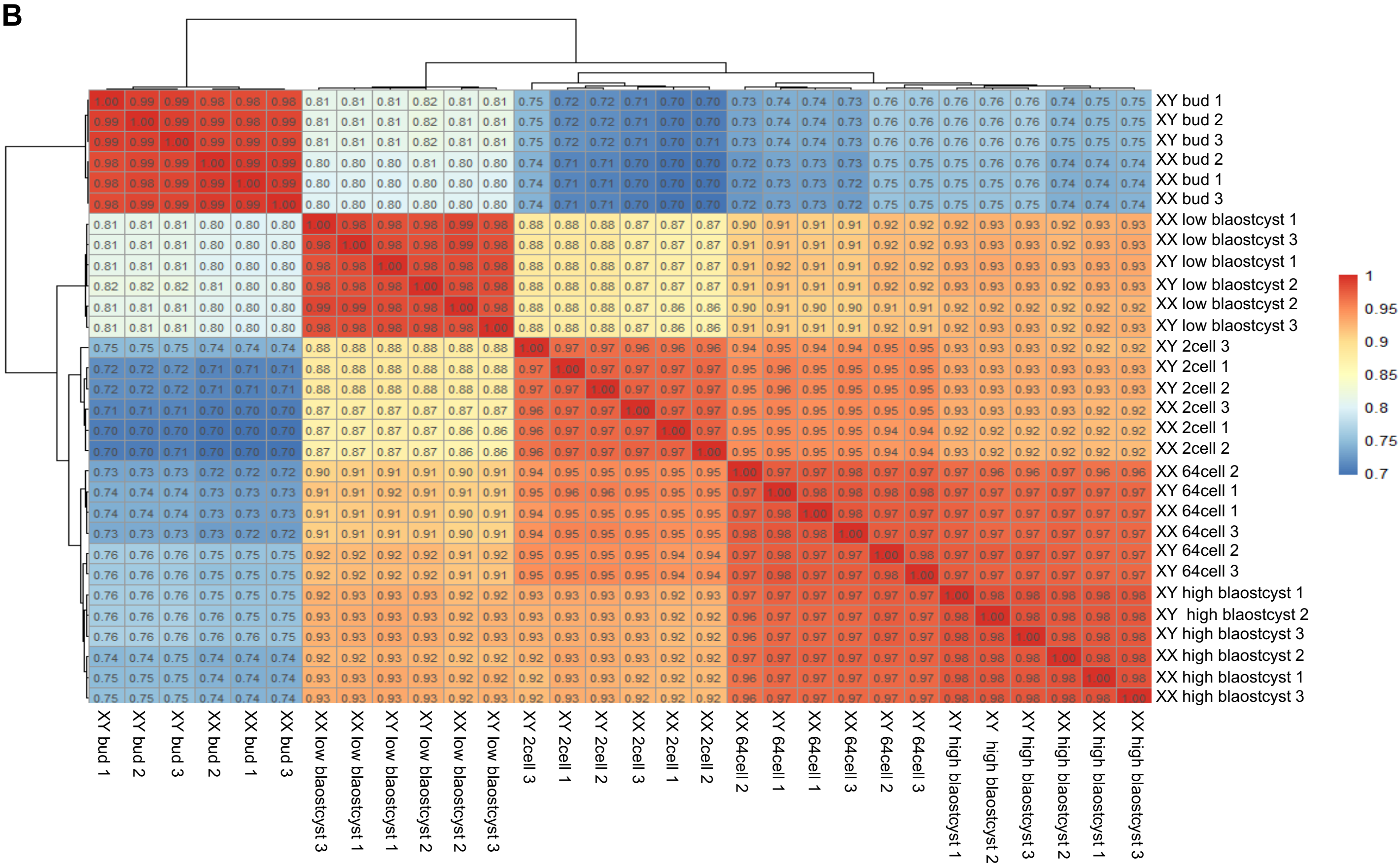

C

sample	maternal	zygotic	sample	maternal	zygotic
xx-2-cell-1	2875	40	xy-2-cell-1	3280	60
xx-2-cell-2	2892	43	xy-2-cell-2	3138	42
xx-2-cell-3	2947	40	xy-2-cell-3	3186	79
xx-64-cell-1	3434	56	xy-64-cell-1	3670	61
xx-64-cell-2	3656	47	xy-64-cell-2	3729	88
xx-64-cell-3	3511	56	xy-64-cell-3	3653	79
xx-high-blastocyst-1	3722	128	xy-high-blastocyst-1	3805	84
xx-high-blastocyst-2	3510	62	xy-high-blastocyst-2	3787	105
xx-high-blastocyst-3	3610	107	xy-high-blastocyst-3	3659	109
xx-low-blastocyst-1	2561	5300	xy-low-blastocyst-1	2864	6077
xx-low-blastocyst-2	2650	5643	xy-low-blastocyst-2	2890	6519
xx-low-blastocyst-3	2610	4814	xy-low-blastocyst-3	3186	7749
xx-bud-1	1404	12280	xy-bud-1	1523	12296
xx-bud-2	1332	11141	xy-bud-2	1633	13770
xx-bud-3	1507	11438	xy-bud-3	1532	13883
