# Supplementary figures and images for: Sexually Dimorphic Gene Expression in X and Y Sperms Instructs Sexual Dimorphism of Embryonic Genome Activation in Yellow Catfish (Pelteobagrus fulvidraco)
Source: Biology (Basel). 2022 Dec 14;11(12):1818. doi: 10.3390/biology11121818 (PMC9775105; doi:10.3390/biology11121818)

A

| Sperm for RNA-seq | Raw reads | Clean reads after trimming | Mapping rate (%) |
|-------------------|-----------|----------------------------|------------------|
| X1                | 56872626  | 55089120                   | 94.61            |
| X2                | 79365106  | 76789194                   | 95.13            |
| X3                | 63236682  | 61315158                   | 94.69            |
| Y1                | 49424868  | 48108746                   | 95.30            |
| Y2                | 51761958  | 54538196                   | 95.43            |
| Y3                | 56414944  | 50417264                   | 95.37            |

B

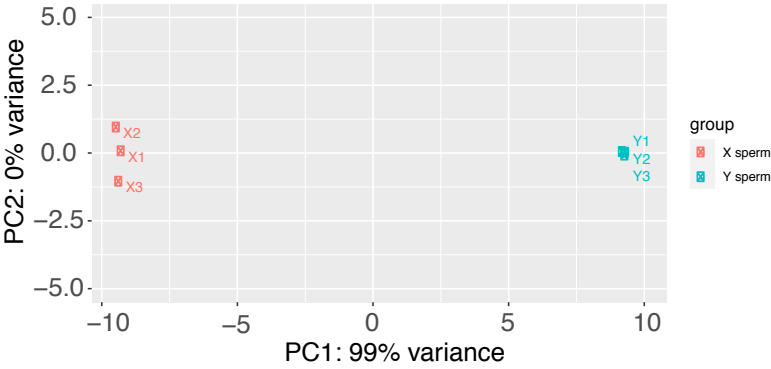

C

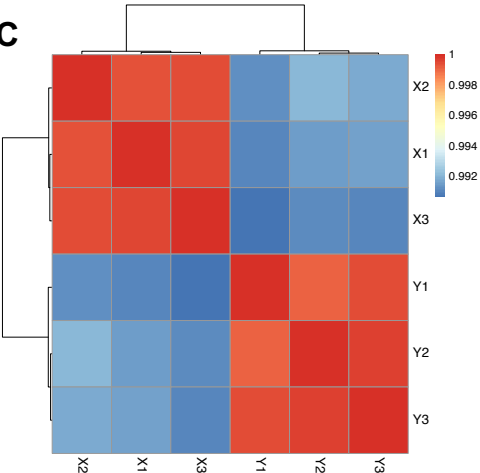

Supplement: Supplementary file 1 [file biology-11-01818-s001.zip › Figure S1.pdf]

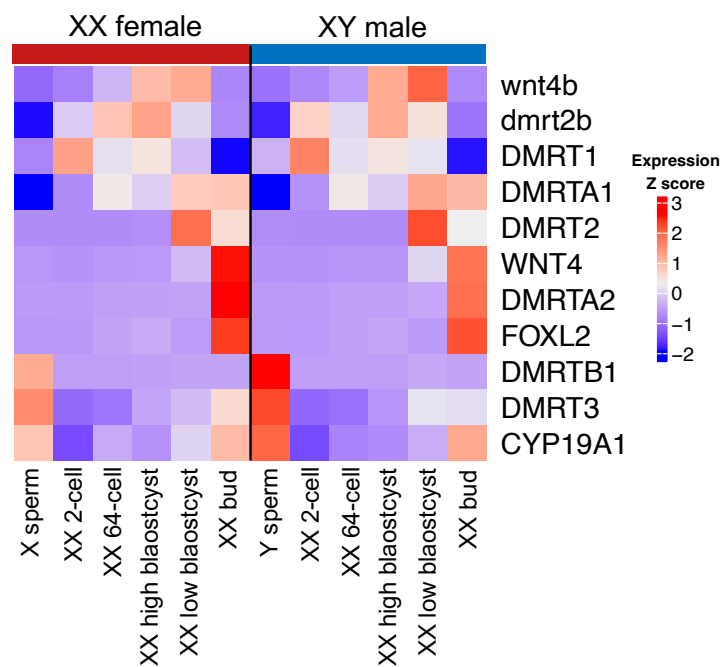

Supplement: Supplementary file 1 [file biology-11-01818-s001.zip › Figure S5.pdf]
